# Neural responsivity to social rewards in autistic female youth

**DOI:** 10.1038/s41398-020-0824-8

**Published:** 2020-06-02

**Authors:** Katherine E. Lawrence, Leanna M. Hernandez, Jeffrey Eilbott, Allison Jack, Elizabeth Aylward, Nadine Gaab, John D. Van Horn, Raphael A. Bernier, Daniel H. Geschwind, James C. McPartland, Charles A. Nelson, Sara J. Webb, Kevin A. Pelphrey, Susan Y. Bookheimer, Mirella Dapretto, on behalf of the GENDAAR Consortium, Elizabeth Aylward, Raphael A. Bernier, Susan Y. Bookheimer, Mirella Dapretto, Nadine Gaab, Daniel H. Geschwind, Allison Jack, James C. McPartland, Charles A. Nelson, Kevin A. Pelphrey, John D. Van Horn, Sara J. Webb, Katy Ankenman, Sarah Corrigan, Dianna Depedro-Mercier, Desiree Guilford, Abha R. Gupta, Zachary Jacokes, Shafali Jeste, Cara M. Keifer, Anna Kresse, Erin Libsack, Jennifer K. Lowe, Erin MacDonnell, Nicole McDonald, Adam Naples, Emily Neuhaus, Catherine A. W. Sullivan, Heidi Tsapelas, Carinna M. Torgerson, Pamela Ventola, Olivia Welker, Julie Wolf

**Affiliations:** 1 Department of Psychiatry and Biobehavioral Sciences, University of California Los Angeles, Los Angeles, USA; 2 Autism & Neurodevelopmental Disorders Institute, The George Washington University School of Medicine and Health Sciences, Washington D.C., USA; 3 Department of Psychology, George Mason University, Fairfax, USA; 4 Center for Integrative Brain Research, Seattle Children’s Research Institute, Seattle, USA; 5 Division of Developmental Medicine, Department of Medicine, Boston Children’s Hospital, Boston, USA; 6 Department of Pediatrics, Harvard Medical School, Boston, USA; 7 Harvard Graduate School of Education, Cambridge, USA; 8 Department of Psychology, University of Virginia, Charlottesville, USA; 9 Department of Psychiatry and Behavioral Sciences, University of Washington, Seattle, USA; 10 Department of Neurology and Center for Neurobehavioral Genetics, University of California Los Angeles, Los Angeles, USA; 11 Department of Pediatrics, Yale School of Medicine, New Haven, USA; 12 Child Study Center, Yale School of Medicine, New Haven, USA; 13 Center on Child Health, Behavior, and Development, Seattle Children’s Research Institute, Seattle, USA; 14 Department of Neurology, University of Virginia, Charlottesville, USA; 15 University of Washington / Seattle Children’s Research Institute, Seattle, USA; 16 University of California, Los Angeles, USA; 17 Harvard University / Boston Children’s Hospital, Boston, USA; 18 George Mason University, Fairfax, USA; 19 Yale University, New Haven, USA; 20 University of Virginia, Charlottesville, USA; 21 University of Southern California, Los Angeles, USA

**Keywords:** Autism spectrum disorders, Neuroscience

## Abstract

Autism is hypothesized to be in part driven by a reduced sensitivity to the inherently rewarding nature of social stimuli. Previous neuroimaging studies have indicated that autistic males do indeed display reduced neural activity to social rewards, but it is unknown whether this finding extends to autistic females, particularly as behavioral evidence suggests that affected females may not exhibit the same reduction in social motivation as their male peers. We therefore used functional magnetic resonance imaging to examine social reward processing during an instrumental implicit learning task in 154 children and adolescents (ages 8–17): 39 autistic girls, 43 autistic boys, 33 typically developing girls, and 39 typically developing boys. We found that autistic girls displayed increased activity to socially rewarding stimuli, including greater activity in the nucleus accumbens relative to autistic boys, as well as greater activity in lateral frontal cortices and the anterior insula compared with typically developing girls. These results demonstrate for the first time that autistic girls do not exhibit the same reduction in activity within social reward systems as autistic boys. Instead, autistic girls display increased neural activation to such stimuli in areas related to reward processing and salience detection. Our findings indicate that a reduced sensitivity to social rewards, as assessed with a rewarded instrumental implicit learning task, does not generalize to affected female youth and highlight the importance of studying potential sex differences in autism to improve our understanding of the condition and its heterogeneity.

## Introduction

Autism is a neurodevelopmental condition characterized by difficulties with social communication, as well as the presence of repetitive behaviors and circumscribed interests^1^. Numerous prior studies have found that autism is associated with reduced attention to and preference for social stimuli as compared with typically developing (TD) controls^2–4^. This reduction in social attention is posited to be in part driven by diminished sensitivity to the intrinsically rewarding nature of social stimuli, which contributes to fewer opportunities for social learning, thereby leading to the social challenges observed in autism^5–8^. Recent functional magnetic resonance imaging (fMRI) studies in primarily male samples have directly tested this hypothesis by examining neural activity in autism when social stimuli are presented as feedback during tasks (e.g., a picture of a smiling face appearing as feedback when participants make a correct choice)^9–17^. This body of work has provided evidence in support of the notion that autistic individuals display reduced neural activation compared with their neurotypical peers in reward-related frontostriatal circuitry and corticolimbic regions during the anticipation and/or receipt of positive social feedback^9,12^. Results from these studies have suggested that such reductions in reward sensitivity may be particularly pronounced for social stimuli^9,10^, although autistic individuals also exhibit altered reward responsivity to non-social stimuli, including corticostriatal hypoactivity to monetary rewards^12,16–18^ and hyperactivity in frontostratial circuits when viewing images which reflect their preferred interests during a rewarded task^15–19^.

Autism is more frequently diagnosed in boys than girls, and all fMRI studies to date investigating reward responsivity in autism have thus included few or no female participants^9–15^. Although the discrepancy in autism prevalence rates between males and females may be exaggerated by sex-specific social and diagnostic factors^20^, the fact that autism is more common among boys is consistently demonstrated, with recent prevalence estimates providing a sex ratio of approximately 4:1 males to females when using registry-based data and approximately 3:1 when using population-based data^21,22^. Importantly, there is evidence for a distinct autism phenotype among affected girls and women, suggesting that previous findings from reward-based fMRI studies in primarily male samples may not generalize to female samples. Autistic females significantly differ from autistic males in their neural activity when processing emotional stories^23^, their patterns of functional and structural brain connectivity^24–27^, their genetic load for autism^28–30^, and their behavioral and cognitive profiles^20,31–35^. Most notably, autistic females and males display different social engagement patterns: autistic girls indicate that they have closer friendships and a greater number of friends than their male counterparts^36–39^. Eye-tracking studies have similarly found that autistic girls exhibit more typical volitional social attention than autistic boys, as well as comparatively reduced attention to objects associated with circumscribed interests^40,41^. In line with such reports, one small self-report study with an approximately equal number of female and male adolescents found that autistic girls displayed significantly greater social motivation than autistic boys as assessed using qualitative interviews^38^.

Importantly, such evidence suggests that findings of reduced social reward sensitivity in autism^5–8^ may not generalize to autistic females. We therefore examined the neural basis of social reward processing in a balanced sample of autistic and TD girls and boys. Specifically, we investigated how social reward processing may differ between autistic girls and boys, as well as between autistic and TD girls. We additionally explored how alterations in such reward processing among autistic youth might be related to individual variability in implicit social learning and core autism traits. By characterizing the neural underpinnings of core autism symptomatology in girls, these analyses improve our understanding of both sex differences in autism and factors which may contribute to the considerable heterogeneity observed among individuals with autism^42,43^.

## Materials and methods

### Participants

Youth (ages 8–17) were recruited from four sites (Harvard Medical School, Seattle Children’s Research Institute, University of California Los Angeles [UCLA], and Yale University) as part of the Gender Exploration of Neurogenetics and Development to Advance Autism Research (GENDAAR) consortium, supported by an NIH Autism Center of Excellence Network. Exclusion criteria for all participants were as follows: premature birth, any known genetic condition (e.g., Fragile X), a history of neurological disorders involving pathology above the brainstem (except uncomplicated non-focal epilepsy), active seizures within the last year, and an inability to comprehend task instructions. For the autism group, inclusion criteria included a prior diagnosis of autism spectrum disorder confirmed by a trained, research-reliable clinician using the Autism Diagnostic Interview, Revised (ADI-R)^44^ and/or Autism Diagnostic Observation Schedule, Second Edition (ADOS-2)^45^. Inter-site reliability was maintained for these measures by having all lead clinicians team double-code one ADI-R and one ADOS-2 every six months, with intra-site reliability maintained by having each site’s lead clinician double-code 10% of assessments. Within the final sample, nearly all autistic youth met criteria on both the ADI-R and the ADOS-2 (*n* = 76/82). A smaller number of participants met criteria on the ADI-R but either were subthreshold on the ADOS-2 by virtue of falling one point short of the total diagnostic cut-off (*n* = 2) or did not receive the ADOS-2 because they were lost to follow-up (*n* = 1); a similarly small number of participants met criteria on the ADOS-2 but either were subthreshold on the ADI-R by virtue of falling one point short of the total diagnostic cut-off (*n* = 1) or did not receive the ADI-R because they were lost to follow-up (*n* = 2). TD participants were required to have no first- or second-degree relatives with autism, no developmental, neurological, or psychiatric disorders, and no evidence of elevated autism traits based on total *t*-scores < 65 on parent-report version of the Social Responsiveness Scale, Second Edition (SRS-2)^46^. After excluding participants with incomplete neuroimaging or IQ data, or excessive motion during scanning, youth were further excluded for being the sibling of another participant in the study. The retained sibling was selected with the goal of minimizing any potential group differences in pubertal development (as measured by the Pubertal Developmental Scale; PDS^47^), site/scanner, motion during the fMRI task (as measured by mean relative motion and the number of censored timepoints) and, separately within each diagnostic group, autism traits (as measured by the SRS-2 for both diagnostic groups and the ADOS-2 for the autistic participants); this selection was done prior to data analysis and prioritized matching participants on autism traits and motion, followed by site/scanner and pubertal development. The final sample included 154 youth: 39 autistic girls, 43 autistic boys, 33 TD girls, and 39 TD boys. Assignment to the female/girl or male/boy group for our analyses was based on parent-report of biological sex designated at birth; no assessment was made of gender identity. As calculated in G*Power 3.1^48^ for a 0.05 significance level, this sample size has 92–95% power to detect large effect sizes (Cohen’s *d* > 0.8) for our between-group ROI analyses. Anonymized data are publicly available for these participants through the National Database for Autism Research (NDAR) under collection ID 2021. Informed assent and consent were obtained from all participants and their legal guardians, and the experimental protocol was approved by the Institutional Review Board at each participating site.

Descriptive statistics and two-tailed *p*-values for our final sample are presented in Table 1; the reported statistical comparisons were completed in R^49^ using *t*-tests, chi-squared tests or their non-parametric equivalent as appropriate. Within both diagnostic groups, girls and boys did not significantly differ on age, general cognitive ability, handedness, household income, mean relative motion, and number of censored fMRI timepoints; there were additionally no significant differences in fMRI task performance as quantified by reaction time (button press speed during social trials), number of correct/incorrect trials (total count of correct and incorrect social trials, respectively), and improvement in task accuracy (change in accuracy between the first and last third of nonrandom social trials) (all *p* > 0.05). Within each diagnostic group there was likewise no significant effect of sex on overall autism traits, as measured by the SRS-2 for both groups and the ADOS-2 for the autism group (all *p* > 0.1). Of particular note, the range of autism traits was similar between autistic girls and boys (ADOS-2 Calibrated Severity Score: 3–10 for girls vs. 3–10 for boys. SRS Total T-Score: 47–90 for girls vs. 54–90 for boys. SRS Total Raw Score: 22–162 for girls vs. 43–146 for boys). Not surprisingly since neither the autistic nor TD sex groups significantly differed on age, girls and boys within each diagnostic group differed in pubertal development (both *p* < 0.01), with girls showing more advanced pubertal development. When testing for demographic differences within each sex, the autism and TD groups did not significantly differ on age, general cognitive ability, handedness, household income, mean relative motion, number of censored fMRI timepoints, and task performance (all *p* > 0.05), with the exception of TD boys displaying higher general cognitive ability scores and lower mean relative motion than autistic boys (both *p* < 0.01). Psychotropic medication information for our sample is presented in Table S1. Notably, the number of participants on medication did not significantly differ between the autistic female and male groups (*p* = 0.9).

**Table 1:** Mean and standard deviation of sample descriptives.

	Autism		TD		F vs. M *P*-values		Autism vs. TD *P*-values	
	Female	Male	Female	Male	Autism	TD	Female	Male
Sample size	39	43	33	39	–	–	–	–
Age (years)	13.23 ± 2.43	13.10 ± 3.10	13.59 ± 2.98	13.31 ± 2.71	0.83	0.67	0.57	0.75
Pubertal development	13.05 ± 3.83^a^	10.68 ± 4.06^b^	13.29 ± 4.26^b^	10.23 ± 3.74	0.009	0.002	0.81	0.61
General conceptual ability	103.38 ± 21.52	103.95 ± 20.16	109.76 ± 16.87	115.33 ± 15.76	0.91	0.15	0.17	0.006
Handedness (R/L)	34/5	41/2	32/1	39/0	0.25	0.46	0.21	0.50
Household income (1/2/3/4/5/6/7/8/9/10)	0/0/1/1/0/1/4/2/3/7 ^g^	0/1/0/1/2/5/4/4/5/7 ^f^	0/0/0/0/1/1/2/7/4/8^d^	1/0/0/0/1/0/3/6/6/10^e^	1.00	0.92	1.00	0.78
Scanner (HT/ST/SP/UT/UP/YT)	1/9/3/9/4/13	5/2/10/12/5/9	4/3/8/5/6/7	3/2/7/10/7/10	0.03	0.83	0.08	0.94
Reaction time (social trials; ms)	800.0 ± 145.8	746.25 ± 190.5	829.1 ± 142.8	801.1 ± 160.9	0.16	0.44	0.40	0.16
# Correct social trials	16.31 ± 2.81	16.21 ± 2.61	16.64 ± 3.31	16.77 ± 2.32	0.87	0.84	0.65	0.31
# Incorrect social trials	17.64 ± 3.24	16.65 ± 2.64	16.55 ± 2.92	16.41 ± 2.76	0.13	0.84	0.14	0.69
Accuracy improvement (social trials; %)	4.87 ± 29.58	−0.73 ± 30.46	15.21 ± 27.28	3.86 ± 27.61	0.40	0.09	0.13	0.48
Mean relative motion (mm)	0.11 ± 0.11	0.13 ± 0.13	0.09 ± 0.07	0.09 ± 0.09	0.49	0.97	0.36	0.009
Timepoints censored	8.77 ± 6.73	10.37 ± 5.68	9.15 ± 7.09	8.00 ± 6.30	0.25	0.47	0.82	0.08
SRS-2 total raw	93.47 ± 31.58^a^	91.93 ± 23.45^c^	18.18 ± 12.33	16.90 ± 13.33	0.81	0.67	<0.001	<0.001
SRS-2 total T-score	76.53 ± 12.10^a^	73.18 ± 9.26^c^	45.48 ± 5.15	43.38 ± 5.55	0.17	0.10	<0.001	<0.001
ADOS-2 calibrated severity score	6.61 ± 1.88^a^	7.33 ± 2.07	–	–	0.11	–	–	–

Handedness: *R* Right, *L* Left. Household Income: 1 = $0–5000, 2 = $5001–10,000, 3 = $10,001–15,000, 4 = $15,001–25,000, 5 = $25,001–35,000, 6 = $35,001–50,000, 7 = $50,001–75,000, 8 = $75,001–100,000, 9 = $100,001–150,000, 10 = Over $150,000. Scanner: *HT* Harvard Trio, *ST* Seattle Trio, *SP* Seattle Prisma, *UT* UCLA Trio, UP UCLA Prisma, *YT* Yale Trio. *TD* typically developing, *F* female. *M* male, *SRS-2* Social Responsiveness Scale, Second Edition, *ADOS-2* Autism Diagnostic Observation Schedule, Second Edition. Superscripts indicate data missing from 1^a^, 2^b^, 3^c^, 10^d^, 12^e^, 14^f^, or 20^g^ subjects.

### fMRI task

**Fig. 1:**
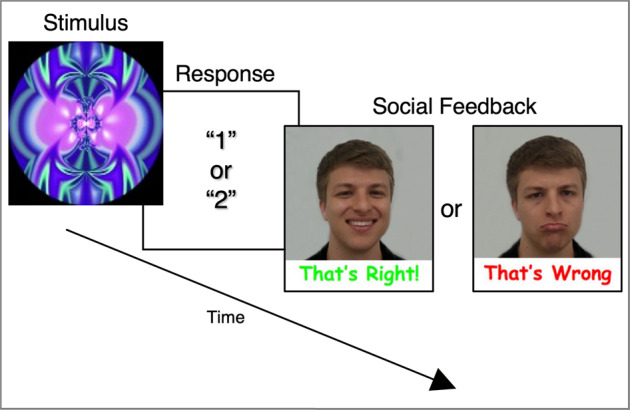
fMRI task. Schematic of an individual social trial within the experimental paradigm.

We used a slightly modified version of the event-related rewarded implicit learning task previously used by our group to investigate reward processing in autistic and TD boys^9^; this task is based on the well-established Weather Prediction Task^50–54^ and was chosen to improve comparability with our prior study^9^. Participants completed several practice trials prior to the scan to ensure they understood task instructions. Briefly, participants were told that abstract images would be presented multiple times over the course of the task, and each time such an image appeared they should guess via button press whether it belonged to “Team 1” or “Team 2”, after which they would receive feedback on their response. Participants thus used trial-and-error over the course of the task to learn which stimuli were associated with which button press, although the implicit learning nature of the task was intentionally not mentioned to subjects. More specifically, each trial started with the presentation of a single abstract fractal-like stimulus. A total of six abstract fractal-like images were included over each administration of the experiment, with four of the six images having a predictive probability of 83%, and the two remaining images having a predictive probability of 50% (i.e., they were associated with a correct response of “1” or “2” for an equal number of trials); this predictive probability distribution was selected to ensure sufficient task difficulty such that participants would rely on implicit learning instead of explicit memorization strategies. While viewing the abstract image presented at the beginning of each trial, participants indicated a response via button press. After 2 sec (s) had elapsed since the trial start, participants received feedback for a duration of 1.25 s. Feedback in the social condition consisted of a smiling male or female face with the text “That’s right!” if the participant had guessed correctly. If the subject’s response was incorrect, the same male or female face with a sad expression was displayed along with the text “That’s wrong” (Fig. 1). In the neutral condition, feedback consisted of the corresponding text “That’s right” or “That’s wrong” and an image of a male or female face displaying a neutral expression. The identity of the male face and the female face were kept constant throughout the experiment. Distinct abstract images were used for the social and neutral trials within each participant; across participants, abstract images were randomly assigned to the social and neutral conditions. After each trial was complete, a blank screen that lasted between 0.73 s and 3.7 s was shown before the start of the next trial. The primary differences between the original and current versions of the task were two-fold^9^. First, we did not use a monetary condition because we were specifically interested in social reward processing, and our initial study using this task found no significant differences in the activity of key reward circuitry (i.e., ventral striatum) during monetary reward processing when comparing autism and typical development. Second, the faces used as feedback in the current task were both male and female, whereas the face used as feedback in the original task was always female; this very minor modification was due to the present study involving both female and male participants, whereas the original sample contained only male youth^55,56^.

### Data acquisition

MRI data were acquired on a Siemens 3T Trio scanner at each site using a 12-channel headcoil or, after scanner upgrades at two sites (Seattle and UCLA), on a Siemens 3T Prisma scanner using a 20-channel headcoil. During the task-based scan (TR = 2000ms, TE = 30 ms, field of view [FOV] = 192 mm, 34 slices, slice thickness = 4 mm, in-plane voxel size = 3 × 3 mm, acquisition time = 5 min; Trio and Prisma parameters were identical), participants viewed stimuli through MR-compatible goggles (Resonance Technology, Inc., Northridge, CA, USA) and indicated their responses on an MR-compatible button box (Resonance Technology, Inc., Northridge, CA, USA). Stimuli presentation and response recording were done using E-Prime 2 (Psychology Software Tools, Inc., Sharpsburg, PA, USA). A T2-weighted high-resolution echo planar scan (Trio: TR = 5000 ms, TE = 34 ms, FOV = 192 mm, 34 slices, slice thickness = 4 mm, in-plane voxel size of 1.5 × 1.5 mm, acquisition time = 1.5 min; Prisma parameters were identical except TE = 35 ms) was also collected for registration purposes. To control for potential between-scanner differences in the multi-site fMRI data, three phantoms (one agar-filled sphere and two human phantoms) traveled to multiple sites. The temporal signal-to-noise ratio (tSNR) was then calculated for each phantom/scanner combination using Analysis of Functional NeuroImages (AFNI)^57^ (Table S2). A linear mixed model including scanner as a fixed effect and phantom as a random effect indicated a significant main effect of scanner on the tSNR calculated from the phantoms (*p* = 0.02). Site/scanner was therefore included as a nuisance covariate in all neuroimaging analyses to statistically control for site/scanner.

### Data analysis

fMRI data underwent standard preprocessing using FMRIB’s Software Library (FSL)^58^, including skull stripping^59^, motion correction^60^, smoothing with a 5 mm full width at half maximum (FWHM) Gaussian kernel, high-pass filtering, and linear registration with 6 degrees of freedom to each subject’s high-resolution matched bandwidth coplanar image, followed by affine registration to the standard MNI template of 152 averaged brains^60,61^; all registrations were visually inspected as part of quality control procedures. For each subject, voxelwise regression analyses were completed using FMRIB’s Improved Linear Model (FILM)^62^. Each trial type was coded as a separate explanatory variable and convolved with a canonical (double-gamma) hemodynamic response function, with the temporal derivative of each trial type likewise included as a regressor. Timepoints which were corrupted by motion were also included as individual regressors to censor them. Specifically, timepoints were censored if their DVARS value was greater than a participant-specific boxplot cut-off equal to the 75th percentile plus 1.5 times the interquartile range as calculated using fsl_motion_outliers.

All group-level analyses assessed social reward processing by examining the task-based contrast “correct social” > “incorrect social” (i.e., happy face with the text “That’s right!” > sad face with the text “That’s wrong”); this allowed for direct comparability with our previous fMRI study which used a virtually identical task and examined this specific contrast to investigate neural responsivity to social rewards in an independent sample of autistic and TD boys^9^. For the current study, our planned key between-group contrasts specifically focused on how autistic girls differed both from autistic boys (female autism vs. male autism) and from their TD peers (female autism vs. female TD); using such pairwise contrasts is in line with previous behavioral and neuroimaging studies examining sex differences in autism^23,33,35^ and also allowed for direct comparison with previous studies which contrasted autistic and TD boys^9,12^. For completeness, we also tested whether autistic boys displayed significant alterations relative to their male counterparts (male autism vs. male TD), as well as whether TD girls and boys differed from each other (female TD vs. male TD). All neuroimaging analyses included the following demographic variables as covariates of non-interest to control for variability within groups and/or significant differences between groups in these variables: site/scanner, demeaned age, demeaned pubertal development (as assessed by the PDS), and demeaned general cognitive ability (as assessed by the DAS-II). For our between-group contrasts, we were primarily interested in the nucleus accumbens (NAcc) due to its importance in reward processing^63–65^ and prior studies demonstrating activity- and connectivity-based alterations of the NAcc in autism^9,66,67^, including NAcc functional connectivity differences between autistic girls and boys^68^. We thus used a region of interest (ROI) approach to examine NAcc activity, where we extracted parameter estimates from each group using a bilateral NAcc mask defined from the Harvard-Oxford Atlas at a threshold of 25% probability^66^. One sample *t*-tests were then completed to determine if each group displayed significant activity when averaging across all of the voxels included in this bilateral ROI, and general linear models with our covariates of non-interest were used to conduct between-group comparisons; residuals from all general linear models were confirmed to meet the assumptions of independence, normality, and constant variance based on visual inspection of residual histograms and residual plots. In addition to our ROI-based analyses, we also conducted exploratory whole-brain analyses using FMRIB’s Local Analyses of Mixed Effects (FLAME 1 + 2) with variance estimated separately for autistic and TD participants. These analyses were limited to gray matter voxels using a prethreshold mask derived from the Harvard-Oxford atlas at a threshold of 25% probability with a small-volume correction for the NAcc due to our specific interest in this structure and the complementary information provided by ROI-based and whole-brain voxelwise analyses. All whole-brain contrasts included our covariates of non-interest and were corrected for multiple comparisons using Gaussian random-field theory in FSL with a voxelwise threshold of *Z* > 2.3 and a corrected cluster threshold of *p* < 0.05; this statistical threshold was chosen to improve comparability with our previous study on social reward responsivity during an essentially identical task in autistic and TD boys^9^. Effect sizes for all significant between-group differences were calculated using extracted parameter estimates and are reported as Cohen’s *d*.

To strengthen the clinical relevance of any significant neural findings, we assessed how significant brain-based differences in social reward processing were associated with individual variability in implicit social learning and overall autistic traits. Specifically, we extracted parameter estimates from those regions which showed significant between-group differences and used a general linear model that included our covariates of non-interest to test whether activity within such brain regions was significantly related to implicit social learning in the autistic or TD participants (i.e., improved accuracy in social trials across the course of the task), parent-reported overall autistic traits in the autism or TD groups (i.e., total raw SRS-2 scores), and clinician-observed overall autistic traits among autistic youth (i.e., ADOS-2 Calibrated Severity Scores). As an example, if there were significant differences between autistic and TD girls in the whole-brain analyses, parameter estimates would be extracted from the significant between-group clusters to quantify neural activity; these measures of brain activity would then be related to implicit learning rate and total autistic traits separately for the female autism and female TD groups. In our completed analyses, behavioral associations were tested for both the ROI-based and whole-brain analyses due to the complementary information provided by these two approaches. Two-tailed *p*-values are reported, with Pearson’s *r* included to convey the magnitude of significant associations.

To assess whether our findings were specific to social reward processing, supplementary analyses were completed examining neural responsivity to non-social rewarding feedback. Specifically, we contrasted brain activity during correct and incorrect neutral trials (which differed in their written feedback but did not differ in social content, as both displayed a neutral facial expression) using the ROI and whole-brain approaches detailed directly above to investigate non-social reward processing (Supplementary Results; Table S3). For completeness, we also conducted additional social reward analyses which examined the main effects of diagnosis and sex as well as their interaction using the NAcc ROI and whole-brain approaches previously described (Supplementary Results; Table S4). Finally, to confirm that our primary, pairwise social reward results were not driven by effects of medication, inclusion of autistic participants who did not meet criteria on both the ADI-R and the ADOS-2, motion confounds, or an interaction between age and group, we completed several supplementary analyses using the parameter estimates extracted from the NAcc ROI and from the significant clusters in our main whole-brain analyses. First, we contrasted neural activity between medicated and unmedicated autistic youth separately within each sex to assess the potential impact of medication (Supplementary Results). Second, we repeated our between-group contrasts of interest on the extracted parameter estimates using a general linear model which was identical to our primary analyses, except that the autistic participants who did not meet criteria on both the ADI-R and the ADOS-2 were excluded, or the model was supplemented with mean relative motion or the interaction between age and group (see Supplementary Results). Results from all additional analyses are presented in the Supplementary Material. Code for all analyses is available upon request.

## Results

### ROI analyses

Descriptive within-group analyses averaging across our bilateral NAcc ROI revealed that autistic girls exhibited significant increases in neural activity to socially rewarding stimuli (*p* = 0.0007), whereas autistic boys did not (*p* = 0.4). Among TD girls, significantly greater mean NAcc activity was similarly observed for “correct social” trials compared with “incorrect social” trials (*p* = 0.008). TD boys demonstrated no significant difference between these two conditions when averaging across the NAcc ROI (*p* = 0.3), although as demonstrated below they did display increased activity to “correct social” trials within a region of the NAcc at the whole-brain level (Fig. S1, Table S5).

**Fig. 2:**
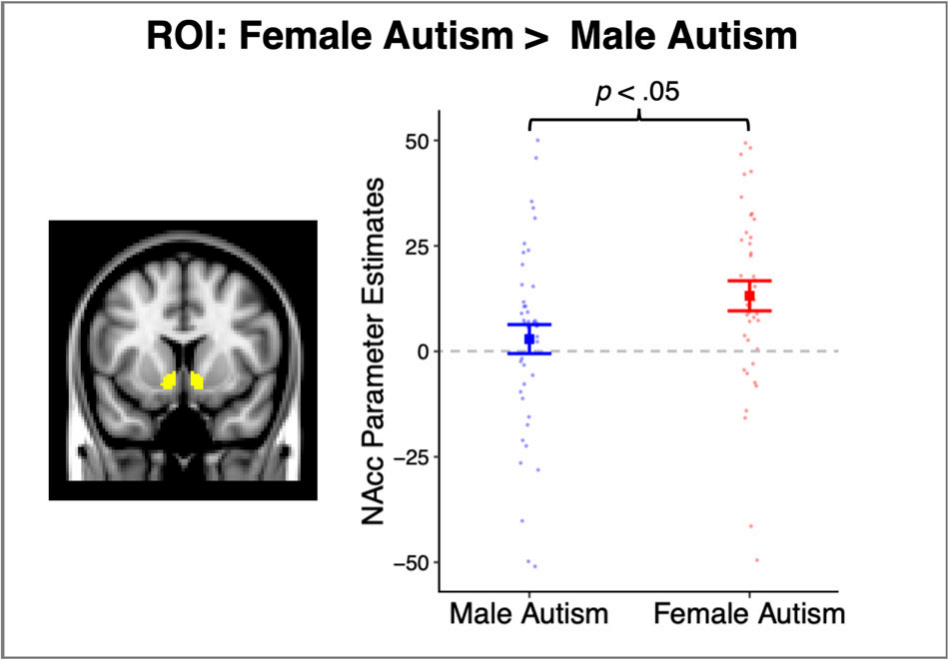
Significant group differences in region of interest (ROI)-based nucleus accumbens (NAcc) activity to social rewards. When averaging across all voxels in the bilateral NAcc region of interest (left), autistic females displayed significantly greater mean activity to social rewards than autistic males (right); error bars are +/− 1 standard error of the mean.

Between-group analyses revealed that autistic girls displayed greater mean bilateral NAcc activity to socially rewarding stimuli than their male counterparts (*p* = 0.03, *d* = 0.53; Fig. 2). In contrast, TD girls and boys did not significantly differ in their NAcc activity to social rewards (*p* = 0.8). We additionally examined whether autistic girls and boys differed from their same-sex TD counterparts and found no significant differences when averaging within the NAcc ROI (both *p* > 0.2). To inform our understanding of how NAcc activity to socially rewarding stimuli is associated with individual differences among autistic youth, we extracted parameter estimates from the NAcc ROI for the female and male autism groups. Neither group exhibited a significant association between this measure of NAcc activity and improved accuracy over the course of the task or the magnitude of core autism traits as measured by the SRS-2 or ADOS-2 (all *p* > 0.05).

### Whole-brain analyses

Descriptive within-group results are presented in Figure S1 and Table S5. Briefly, autistic girls and boys both displayed greater activity to “correct social” trials than “incorrect social” trials in a number of cortical regions, including the ventromedial prefrontal cortex (vmPFC), the medial prefrontal cortex (mPFC), and the anterior cingulate cortex (ACC). Autistic girls also exhibited widespread activity to socially rewarding stimuli in a range of additional cortical and subcortical areas, including the NAcc, the insula, and lateral frontal cortex. With regards to the control groups, TD girls and TD boys both displayed increased activity to socially rewarding stimuli within the NAcc.

**Fig. 3:**
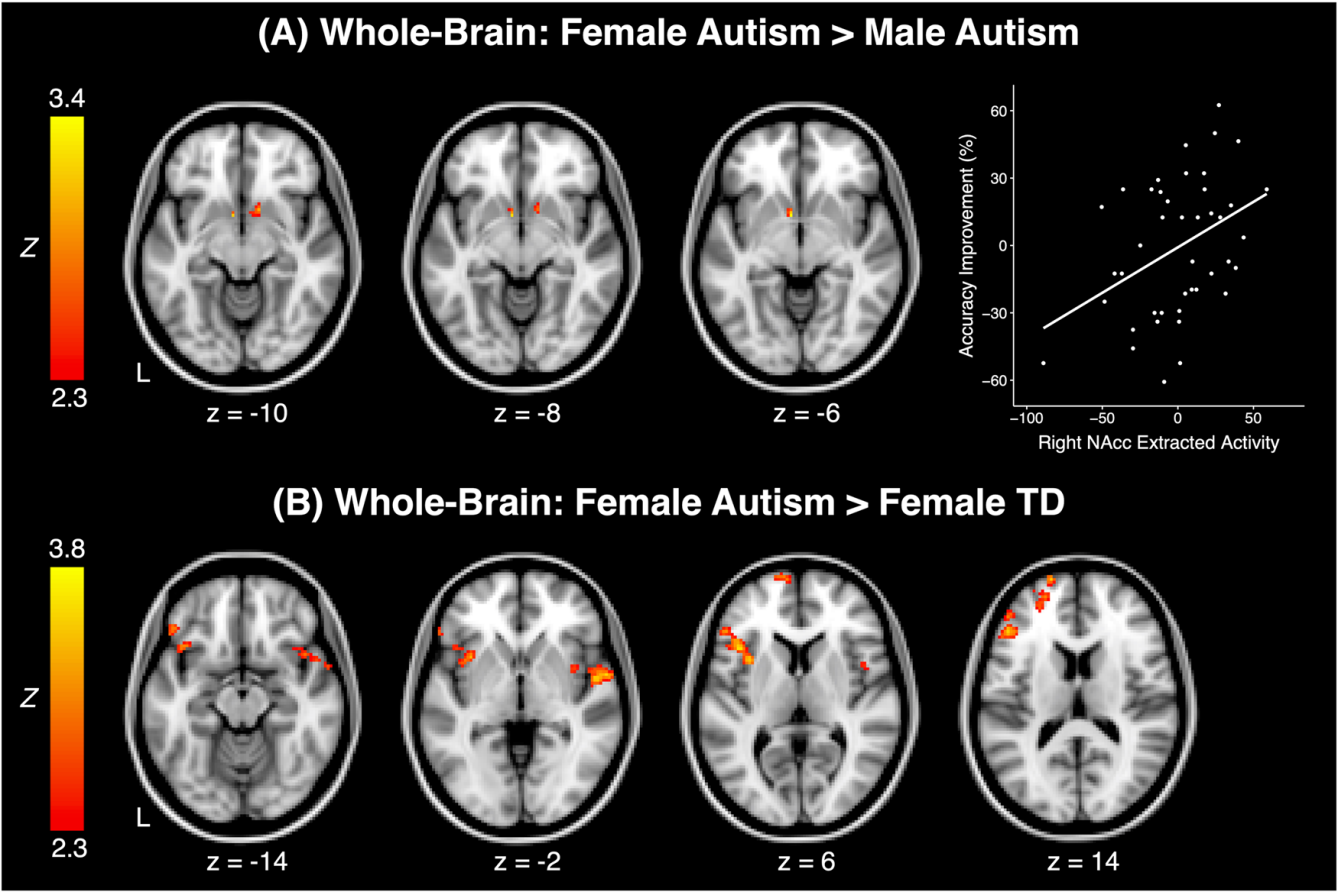
Significant group differences in whole-brain activity to social rewards and relationship with behavior. **a** Areas in which autistic girls exhibited significantly greater activity to socially rewarding stimuli than autistic boys in the whole-brain analyses (left). In the group of autistic boys, increased activity to social rewards in the right NAcc cluster was related to improved accuracy over the course of the task (i.e., greater implicit learning) (right); plotted values reflect mean parameter estimates extracted from the significant whole-brain cluster located in the right NAcc. **b** Regions in which autistic girls displayed significant hyperactivity to social rewards compared with TD girls in the whole-brain analyses. NAcc: nucleus accumbens; TD: typically developing; L: Left.

When directly comparing girls and boys within each diagnostic group, autistic girls displayed greater neural activity than autistic boys in both the left and the right NAcc during social rewards (Fig. 3a; Table 2). Unlike autistic youth, there were no significant sex differences among TD youth in their neural responsivity to socially rewarding stimuli. Autistic girls also exhibited greater neural activity to social rewards than TD girls (Fig. 3b; Table 2); such hyperactivity among autistic girls was primarily visible within lateral frontal regions, including the ventrolateral prefrontal cortex (vlPFC) and the lateral orbitofrontal cortex (OFC), in addition to the anterior insula and other frontal and temporal regions (Table 2). Autistic boys did not significantly differ in whole-brain analyses from their TD counterparts.

**Table 2:** Peak coordinates for altered whole-brain activity to social rewards in autistic females.

Region	L/R	Max *Z*	MNI Peak (mm)			Sig # Voxels
			x	y	z	
Female autism > Male autism						
*Left nucleus accumbens cluster, d* = 0.41						
Accumbens	L	3.40	−6	6	−6	11
*Right nucleus accumbens cluster, d* = 0.78						
Accumbens	R	3.02	12	10	−10	12
Female autism > Female TD						
*Left frontal/insular cluster, d* = 0.87						
Orbital frontal cortex	L	3.09	−40	20	−14	33
Inferior frontal gyrus	L	3.70	−52	28	4	59
Frontal pole	L	3.35	−48	40	12	28
Frontal operculum cortex	L	3.62	−40	18	6	35
Insula	L	3.57	−34	12	4	51
*Right insular/temporal cluster, d* = 0.89						
Insula	R	2.83	40	12	−12	35
Planum polare	R	3.14	54	0	−2	11
Temporal pole	R	3.44	50	10	−10	43
Central opercular cortex	R	3.02	46	6	4	26
*Left frontal cluster, d* = 0.80						
Frontal pole	L	3.61	−18	68	10	370

Regions were labeled using the Harvard-Oxford atlas at a 50% probabilistic threshold. Left/Right masks excluded the midline, and regions were only listed if they included 10 or more voxels displaying a significant between-group activation difference.

*TD* typically developing, *L* left, *R* right, *MNI* Montreal Neurological Institute.

To examine the relationship between social reward responsivity and individual variability in autism traits and task performance, we extracted parameter estimates from those clusters which displayed a significant difference between autistic girls and boys, in addition to extracting parameter estimates from those clusters which significantly differed between autistic girls and their TD counterparts. No significant associations were found among autistic or TD girls between extracted neural activity and task improvement or autism characteristics (all *p* > 0.1). Likewise, no significant relationship was seen in autistic boys between activity and core autism traits as assessed by the SRS-2 or ADOS-2 (both *p* > 0.05). However, autistic boys displayed a significant relationship between activity within the right NAcc and their ability to implicitly learn from social trials, such that greater social reward activity was associated with greater task-based implicit learning (*p* = 0.04, *r* = 0.43; Fig. 2b).

## Discussion

In the current study, we analyzed a sample of youth between the ages of 8 and 17 years old to investigate how autistic girls process socially rewarding stimuli, and how their neural activity to social rewards may differ from autistic boys and TD girls. Autistic girls exhibited significantly greater neural responsivity to socially rewarding stimuli (i.e., a smiling face) than their male counterparts, reflecting that autistic girls displayed increased neural activation to social rewards but autistic boys did not. One account for the social challenges present in autism is that these are driven by reduced sensitivity to the rewarding nature of social stimuli, which subsequently leads to fewer social learning opportunities and the emergence of the social difficulties characteristic of autism^7,8^. Prior neuroimaging studies in male samples have supported this notion, demonstrating that autistic boys exhibit hypoactivity to positive social stimuli in frontostriatal and limbic circuitry such as the ventral striatum and the amygdala^9,12^, as well as additional corticostriatal and limbic alterations in response to neutral or negative social stimuli^11,13,14,16^, or when contrasting social stimuli with non-social stimuli^10,15^. We replicate these prior findings in the current study with an independent sample. That is, as with previous analyses focusing on positive social stimuli, autistic boys in the current study did not significantly activate the ventral striatum in response to social rewards at the whole-brain level, even though TD boys did. Notably, reduced NAcc activity among our male autism group was significantly related to reduced implicit learning. Much of social learning in the real world occurs without explicit instruction^69^, and these results among autistic boys thus lend novel support to the proposal that diminished social reward sensitivity contributes to a relative lack of social learning, which may ultimately contribute to the core social challenges present in autism.

Although our findings and those of other prior studies lend support to the hypothesis that alterations in social reward sensitivity are related to autism in males^2,4,16^, no neuroimaging studies to date have investigated whether reduced reward responsivity may also explain core autism traits among affected females. Importantly, our results suggest that this may not be the case for autistic girls, as they displayed significantly *increased* neural activity to social rewards despite displaying no differences in activity related to rewards which did not differ in social content. This increased responsiveness to social rewards among autistic girls included greater NAcc activity compared with autistic boys, as well as greater activity within the lateral OFC, vlPFC, and anterior insula relative to TD girls. The anterior insula activates during reward-related tasks^70–74^ and is one of the hubs of the salience network, which plays a role in detecting and coordinating a neural and behavioral response to salient stimuli^75,76^. It is therefore possible that socially rewarding stimuli may possess increased salience to autistic females. With regards to the other regions which exhibited increased activity among autistic girls, the vlPFC and OFC are known to play a role in reversal learning and to encode reward-related characteristics, such as the subjective value and probability of rewards^77–83^. The NAcc has similarly been directly implicated in reward learning and processing. Among neurotypical individuals, neuroimaging analyses have shown that the NAcc consistently activates during the anticipation and receipt of a wide variety of rewards, including monetary and social rewards^64,65,70–74,84^. Animal studies have additionally demonstrated that the NAcc is associated with reward-related hedonic pleasure through the opioid and endocannabinoid systems, as well as incentive motivation and prediction error-based learning through the mesolimbic dopamine pathway^63–65^. Our findings thus suggest that autistic girls may be characterized by an increased motivation to obtain positive social feedback and, furthermore, that these girls’ neural circuitry may be more fine-tuned to learn from such stimuli; this pattern of results is also in line with a recent fMRI study which demonstrated that, unlike autistic men, autistic women do not exhibit hypoactivation of social brain areas^85^. Notably, the current finding of greater neural sensitivity to social rewards in autistic girls may be one biological mechanism for the reduced prevalence of autism among females and for the sex differences in friendship patterns among youth with autism^22,36–38^. Such increased sensitivity to socially rewarding stimuli among autistic girls may also have implications for the use and personalization of reinforcement-based interventions. By suggesting that alterations in social reward responsivity may be less relevant for the etiology of autism in girls, our findings also underscore a need for studies to examine additional factors which may contribute to the development of autism in girls; these studies could, for instance, assess the hypothesized importance of early differences in sensory processing to the development of autism^86^. Such studies would also improve our understanding of the non-social characteristics associated with autism, including sensory overresponsivity, challenges with executive function, and strengths in systemizing^7,87–90^.

Future work should address the limitations of the current study. Such investigations could separately examine the anticipation and receipt of social rewards to test whether these two stages of reward processing are differentially affected among autistic females and males. These experiments could also use stimuli which are more naturalistic, such as dynamic videoclips of social praise instead of the static images used here^15^. Additionally, several prior studies have suggested that autistic boys may be characterized by increased reward responsivity to stimuli related to their circumscribed interests^15,16,18,19^. Future analyses should investigate whether this is also true among autistic girls, particularly as autistic females may exhibit lower levels of circumscribed interests and repetitive behaviors than their male counterparts^32,35,91^. Biological sex assigned at birth also does not always reflect individuals’ gender identity^92^. Future studies should thus explicitly assess gender identity, particularly as autism may be associated with increased rates of gender dysphoria^93^; these analyses could help clarify the respective contributions of gender and biological sex to patterns of altered reward processing in autism. With sufficiently large sample sizes, such studies could also specifically assess the replicability of the current results in an independent sample and furthermore allow for a more comprehensive understanding of the potential main effects of sex/gender and diagnosis, as well as their interaction. Lastly, in the current study there were no significant differences in task performance or overall autistic traits between affected girls and boys, and more fine-grained assessments of implicit learning and autism characteristics were not collected. However, prior work suggests that autistic girls and boys significantly differ in their cognitive and behavioral profiles including, importantly, their level of social motivation^20,31–35,38^. In the future, it will thus be important to collect in-depth measurements of implicit learning, as well detailed self-report and clinical assessments of social motivation, to further characterize the association between neural responsivity to social rewards and individual differences in behavior^38,94,95^.

Taken together, our results are the first to demonstrate that autistic girls display increased responsivity to socially rewarding stimuli, unlike what has been previously reported in autistic males. These findings thus indicate that autistic females may not display the same reductions in social motivation as autistic males and underscore the importance of considering sex when characterizing the mechanisms that may give rise to autism and the considerable heterogeneity associated with this condition.

## Supplementary information

Supplemental Material

## References

[1] American Psychiatric Association. (2013). Diagnostic and Statistical Manual of Mental Disorders..

[2] Dubey I, Ropar D, Hamilton AF (2015). Measuring the value of social engagement in adults with and without autism. Mol. Autism.

[3] Chita-Tegmark M (2016). Social attention in ASD: A review and meta-analysis of eye-tracking studies. Res Dev. Disabil..

[4] Ruta L (2017). Reduced preference for social rewards in a novel tablet based task in young children with autism spectrum disorders. Sci. Rep..

[5] Schultz RT (2005). Developmental deficits in social perception in autism: the role of the amygdala and fusiform face area. Int J. Dev. Neurosci..

[6] Dawson G (2005). Neurocognitive and electrophysiological evidence of altered face processing in parents of children with autism: implications for a model of abnormal development of social brain circuitry in autism. Dev. Psychopathol..

[7] Chevallier C, Kohls G, Troiani V, Brodkin ES, Schultz RT (2012). The social motivation theory of autism. Trends Cogn. Sci..

[8] Dawson G, Bernier R, Ring RH (2012). Social attention: a possible early indicator of efficacy in autism clinical trials. J. Neurodev. Disord..

[9] Scott-Van Zeeland AA, Dapretto M, Ghahremani DG, Poldrack RA, Bookheimer SY (2010). Reward processing in autism. Autism Res.

[10] Delmonte S (2012). Social and monetary reward processing in autism spectrum disorders. Mol. Autism.

[11] Dichter GS, Richey JA, Rittenberg AM, Sabatino A, Bodfish JW (2012). Reward circuitry function in autism during face anticipation and outcomes. J. Autism Dev. Disord..

[12] Kohls G (2013). Reward system dysfunction in autism spectrum disorders. Soc. Cogn. Affect Neurosci..

[13] Choi US (2015). Abnormal brain activity in social reward learning in children with autism spectrum disorder: an fMRI study. Yonsei Med. J..

[14] Damiano CR (2015). Neural mechanisms of negative reinforcement in children and adolescents with autism spectrum disorders. J. Neurodev. Disord..

[15] Kohls G, Antezana L, Mosner MG, Schultz RT, Yerys BE (2018). Altered reward system reactivity for personalized circumscribed interests in autism. Mol. Autism.

[16] Clements CC (2018). Evaluation of the social motivation hypothesis of autism: a systematic review and meta-analysis. JAMA Psychiatry.

[17] Dichter GS, Damiano CA, Allen JA (2012). Reward circuitry dysfunction in psychiatric and neurodevelopmental disorders and genetic syndromes: animal models and clinical findings. J. Neurodev. Disord..

[18] Dichter GS (2012). Reward circuitry function in autism spectrum disorders. Soc. Cogn. Affect Neurosci..

[19] Cascio CJ (2014). Affective neural response to restricted interests in autism spectrum disorders. J. Child Psychol. Psychiatry.

[20] Lai MC, Lombardo MV, Auyeung B, Chakrabarti B, Baron-Cohen S (2015). Sex/gender differences and autism: setting the scene for future research. J. Am. Acad. Child Adolesc. Psychiatry.

[21] Loomes R, Hull L, Mandy WPL (2017). What is the male-to-female ratio in autism spectrum disorder? A systematic review and meta-analysis. J. Am. Acad. Child Adolesc. Psychiatry.

[22] Baio J (2018). Prevalence of Autism Spectrum Disorder Among Children Aged 8 Years — Autism and Developmental Disabilities Monitoring Network, 11 Sites, United States, 2014. MMWR Surveill. Summ..

[23] Schneider K (2013). Evidence for gender-specific endophenotypes in high-functioning autism spectrum disorder during empathy. Autism Res..

[24] Nordahl CW (2015). Sex differences in the corpus callosum in preschool-aged children with autism spectrum disorder. Mol. Autism.

[25] Alaerts K, Swinnen SP, Wenderoth N (2016). Sex differences in autism: a resting-state fMRI investigation of functional brain connectivity in males and females. Soc. Cogn. Affect Neurosci..

[26] Lai MC (2017). Imaging sex/gender and autism in the brain: etiological implications. J. Neurosci. Res.

[27] Lawrence, K. E. et al. Sex differences in functional connectivity of the salience, default mode, and central executive networks in youth with ASD. *Cereb. Cortex*10.1093/cercor/bhaa105 (2020). 10.1093/cercor/bhaa105 PMC7391269 32350530

[28] Werling DM, Geschwind DH (2013). Sex differences in autism spectrum disorders. Curr. Opin. Neurol..

[29] Werling DM (2016). The role of sex-differential biology in risk for autism spectrum disorder. Biol. Sex. Differ..

[30] Ferri SL, Abel T, Brodkin ES (2018). Sex differences in autism spectrum disorder: a review. Curr. Psychiatry Rep..

[31] Baron-Cohen S (2014). Attenuation of typical sex differences in 800 adults with autism vs. 3,900 controls. PLoS ONE.

[32] Frazier TW, Georgiades S, Bishop SL, Hardan AY (2014). Behavioral and cognitive characteristics of females and males with autism in the Simons Simplex Collection. J. Am. Acad. Child Adolesc. Psychiatry.

[33] Hiller RM, Young RL, Weber N (2014). Sex differences in autism spectrum disorder based on DSM-5 criteria: evidence from clinician and teacher reporting. J. Abnorm Child Psychol..

[34] Hull L, Mandy W, Petrides KV (2017). Behavioural and cognitive sex/gender differences in autism spectrum condition and typically developing males and females. Autism.

[35] Knutsen J, Crossman M, Perrin J, Shui A, Kuhlthau K (2018). Sex differences in restricted repetitive behaviors and interests in children with autism spectrum disorder: an Autism Treatment Network study. Autism.

[36] Dean M (2014). The peer relationships of girls with ASD at school: comparison to boys and girls with and without ASD. J. Child Psychol. Psychiatry.

[37] Head AM, McGillivray JA, Stokes MA (2014). Gender differences in emotionality and sociability in children with autism spectrum disorders. Mol. Autism.

[38] Sedgewick F, Hill V, Yates R, Pickering L, Pellicano E (2016). Gender differences in the social motivation and friendship experiences of autistic and non-autistic adolescents. J. Autism Dev. Disord..

[39] Sedgewick F, Hill V, Pellicano E (2019). ‘It’s different for girls’: gender differences in the friendships and conflict of autistic and neurotypical adolescents. Autism.

[40] Harrop C (2018). Sex differences in social attention in autism spectrum disorder. Autism Res..

[41] Harrop C (2018). Circumscribed interests and attention in autism: the role of biological sex. J. Autism Dev. Disord..

[42] Lombardo MV, Lai MC, Baron-Cohen S (2019). Big data approaches to decomposing heterogeneity across the autism spectrum. Mol. Psychiatry.

[43] Hong, S.-K. et al. Towards neurosubtypes in autism. *Biol. Psychiatry*10.1016/j.biopsych.2020.03.022 (2020). 10.1016/j.biopsych.2020.03.022 32553193

[44] Lord C, Rutter M, Le Couteur A (1994). Autism Diagnostic Interview-Revised: a revised version of a diagnostic interview for caregivers of individuals with possible pervasive developmental disorders. J. Autism Dev. Disord..

[45] Lord C, DiLavore PC, Gotham K (2012). Autism Diagnostic Observation Schedule..

[46] Constantino JN, Gruber CP (2012). Social Responsiveness Scale–Second Edition (SRS-2)..

[47] Carskadon MA, Acebo C (1993). A self-administered rating scale for pubertal development. J. Adolesc. Health.

[48] Faul F, Erdfelder E, Buchner A, Lang AG (2009). Statistical power analyses using G*Power 3.1: tests for correlation and regression analyses. Behav. Res Methods.

[49] R Core Team. (2016). R: A language and environment for statistical computing..

[50] Knowlton BJ, Squire LR, Gluck MA (1994). Probabilistic classification learning in amnesia. Learn Mem..

[51] Poldrack RA (2001). Interactive memory systems in the human brain. Nature.

[52] Moody TD, Bookheimer SY, Vanek Z, Knowlton BJ (2004). An implicit learning task activates medial temporal lobe in patients with Parkinson’s disease. Behav. Neurosci..

[53] Kelmendi B (2016). Probing implicit learning in obsessive-compulsive disorder: moderating role of medication on the weather prediction task. J. Obsessive Compuls. Relat. Disord..

[54] Labouliere CD, Terranova K, Steinglass J, Marsh R (2016). Implicit learning on a probabilistic classification task in adults and adolescents with Bulimia Nervosa. J. Psychiatr. Res.

[55] Aharon I (2001). Beautiful faces have variable reward value: fMRI and behavioral evidence. Neuron.

[56] Winston JS, O’Doherty J, Kilner JM, Perrett DI, Dolan RJ (2007). Brain systems for assessing facial attractiveness. Neuropsychologia.

[57] Cox RW (1996). AFNI: software for analysis and visualization of functional magnetic resonance neuroimages. Comput. Biomed. Res..

[58] Smith SM (2004). Advances in functional and structural MR image analysis and implementation as FSL. Neuroimage.

[59] Smith SM (2002). Fast robust automated brain extraction. Hum. Brain Mapp..

[60] Jenkinson M, Bannister P, Brady M, Smith S (2002). Improved optimization for the robust and accurate linear registration and motion correction of brain images. Neuroimage.

[61] Jenkinson M, Smith S (2001). A global optimisation method for robust affine registration of brain images. Med. Image Anal..

[62] Woolrich MW, Ripley BD, Brady M, Smith SM (2001). Temporal autocorrelation in univariate linear modeling of FMRI data. Neuroimage.

[63] Berridge KC, Robinson TE, Aldridge JW (2009). Dissecting components of reward: ‘liking’, ‘wanting’, and learning. Curr. Opin. Pharm..

[64] Daniel R, Pollmann S (2014). A universal role of the ventral striatum in reward-based learning: evidence from human studies. Neurobiol. Learn Mem..

[65] Fareri DS, Delgado MR (2014). Social rewards and social networks in the human brain. Neuroscientist.

[66] Hernandez LM (2017). Additive effects of oxytocin receptor gene polymorphisms on reward circuitry in youth with autism. Mol. Psychiatry.

[67] Supekar K (2018). Deficits in mesolimbic reward pathway underlie social interaction impairments in children with autism. Brain.

[68] Hernandez LM (2020). Imaging-genetics of sex differences in ASD: distinct effects of OXTR variants on brain connectivity. Transl. Psychiatry.

[69] Frith CD (2008). Social cognition. Philos. Trans. R. Soc. Lond. B Biol. Sci..

[70] Liu X, Hairston J, Schrier M, Fan J (2011). Common and distinct networks underlying reward valence and processing stages: a meta-analysis of functional neuroimaging studies. Neurosci. Biobehav Rev..

[71] Diekhof EK, Kaps L, Falkai P, Gruber O (2012). The role of the human ventral striatum and the medial orbitofrontal cortex in the representation of reward magnitude - an activation likelihood estimation meta-analysis of neuroimaging studies of passive reward expectancy and outcome processing. Neuropsychologia.

[72] Sescousse G, Caldu X, Segura B, Dreher JC (2013). Processing of primary and secondary rewards: a quantitative meta-analysis and review of human functional neuroimaging studies. Neurosci. Biobehav. Rev..

[73] Silverman MH, Jedd K, Luciana M (2015). Neural networks involved in adolescent reward processing: an activation likelihood estimation meta-analysis of functional neuroimaging studies. Neuroimage.

[74] Oldham S (2018). The anticipation and outcome phases of reward and loss processing: a neuroimaging meta-analysis of the monetary incentive delay task. Hum. Brain Mapp..

[75] Uddin LQ, Menon V (2009). The anterior insula in autism: under-connected and under-examined. Neurosci. Biobehav. Rev..

[76] Menon V, Uddin LQ (2010). Saliency, switching, attention and control: a network model of insula function. Brain Struct. Funct..

[77] Tobler PN, Christopoulos GI, O’Doherty JP, Dolan RJ, Schultz W (2009). Risk-dependent reward value signal in human prefrontal cortex. Proc. Natl Acad. Sci. USA.

[78] Rygula R, Walker SC, Clarke HF, Robbins TW, Roberts AC (2010). Differential contributions of the primate ventrolateral prefrontal and orbitofrontal cortex to serial reversal learning. J. Neurosci..

[79] Hampshire A, Chaudhry AM, Owen AM, Roberts AC (2012). Dissociable roles for lateral orbitofrontal cortex and lateral prefrontal cortex during preference driven reversal learning. Neuroimage.

[80] Rich EL, Wallis JD (2014). Medial-lateral organization of the orbitofrontal cortex. J. Cogn. Neurosci..

[81] Dalton GL, Wang NY, Phillips AG, Floresco SB (2016). Multifaceted contributions by different regions of the orbitofrontal and medial prefrontal cortex to probabilistic reversal learning. J. Neurosci..

[82] Kaskan PM (2017). Learned value shapes responses to objects in frontal and ventral stream networks in Macaque monkeys. Cereb. Cortex.

[83] Rudebeck PH, Saunders RC, Lundgren DA, Murray EA (2017). Specialized representations of value in the orbital and ventrolateral prefrontal cortex: desirability versus availability of outcomes. Neuron.

[84] Wang KS, Smith DV, Delgado MR (2016). Using fMRI to study reward processing in humans: past, present, and future. J. Neurophysiol..

[85] Lai MC (2019). Neural self-representation in autistic women and association with ‘compensatory camouflaging’. Autism.

[86] Robertson CE, Baron-Cohen S (2017). Sensory perception in autism. Nat. Rev. Neurosci..

[87] Lai M-C, Lombardo MV, Baron-Cohen S (2014). Autism. Lancet.

[88] Ben-Sasson A (2009). A meta-analysis of sensory modulation symptoms in individuals with autism spectrum disorders. J. Autism Dev. Disord..

[89] Lai CLE (2017). Meta-analysis of neuropsychological measures of executive functioning in children and adolescents with high-functioning autism spectrum disorder. Autism Res..

[90] Baron-Cohen S, Ashwin E, Ashwin C, Tavassoli T, Chakrabarti B (2009). Talent in autism: hyper-systemizing, hyper-attention to detail and sensory hypersensitivity. Philos. Trans. R. Soc. Lond. B Biol. Sci..

[91] Tillmann J (2018). Evaluating sex and age differences in ADI-R and ADOS scores in a large european multi-site sample of individuals with autism spectrum disorder. J. Autism Dev. Disord..

[92] Mueller SC, De Cuypere G, T’Sjoen G (2017). Transgender research in the 21st century: a selective critical review from a neurocognitive perspective. Am. J. Psychiatry.

[93] George R, Stokes MA (2018). Gender identity and sexual orientation in autism spectrum disorder. Autism.

[94] Kazdin AE (1989). Evaluation of the Pleasure Scale in the assessment of anhedonia in children. J. Am. Acad. Child Adolesc. Psychiatry.

[95] Gooding DC, Pflum MJ, Fonseca-Pedero E, Paino M (2016). Assessing social anhedonia in adolescence: the ACIPS-A in a community sample. Eur. Psychiatry.

