# Supplementary material for: Neural responsivity to social rewards in autistic female youth
Source: Transl Psychiatry. 2020 Jun 2;10:178. doi: 10.1038/s41398-020-0824-8 (PMC7266816; doi:10.1038/s41398-020-0824-8)
Supplement: Supplementary file 1 — Supplemental Material [file 41398_2020_824_MOESM1_ESM.docx]

**Supplementary Material**

*Supplementary Results*

When examining activity to rewards which did not differ in social content (i.e., positive vs. negative written feedback with the same neutral face), no between-group differences emerged in activity when averaging across the bilateral NAcc ROI. Similarly, whole-brain patterns of activity to positive vs. negative written feedback in the context of the same neutral face (i.e., non-social rewards) did not significantly differ between autistic girls and boys, between TD girls and boys, or between autistic and TD girls; autistic boys exhibited significantly more activity to these non-social rewards than TD boys in the mPFC (Table S3). Analyses investigating the main effects of diagnosis and sex on social reward processing, as well as the interaction between diagnosis and sex, revealed no significant main effects or interaction in the NAcc ROI (all *p* > 0.05). There was a main effect of diagnosis in the whole-brain analyses, such that autistic youth exhibited increased activity to socially rewarding stimuli compared with TD youth in medial and lateral frontal regions (Table S4); the lateral frontal regions largely overlapped with significant clusters found when contrasting autistic and TD girls (Figure 3, Table 2), suggesting this finding was driven by the female autistic youth. A main effect of sex was shown in the whole-brain analyses, such that girls displayed greater social reward activity than boys in the precuneus and lateral occipital cortex (Table S4); these areas did not overlap with the significant differences seen between autistic girls and boys (Figure 3, Table 2), suggesting that sex differences in autism are not a function of participant sex more generally. There was no statistically significant interaction in the whole-brain analyses.

With regards to the impact of medication on our primary pairwise social rewards findings, there were no significant differences between medicated autistic youth and their same-sex unmedicated autistic counterparts when extracting parameter estimates from the NAcc ROI or from the significant clusters in the whole-brain analyses (all *p* > 0.1). Our pairwise between-group social reward findings also remained highly similar when examining extracted parameter estimates after excluding autistic participants who did not meet criteria on both the ADI-R and the ADOS-2, or after including mean relative motion as a covariate of non-interest. In both cases, autistic girls continued to display greater ROI-based NAcc activity than autistic boys, as well as greater right NAcc activity in the whole-brain analyses (both *p* < 0.05); autistic girls likewise continued to exhibit hyperactivity of frontal, insular, and temporal regions compared to TD girls, including the lateral OFC, vlPFC, and anterior insula (all *p* *<* .01). Lastly, there was no significant interaction between group and age when examining activity to socially rewarding stimuli (all *p* > 0.2).

*Supplementary Tables*

Table S1: Psychotropic Medication Usage

|  | Autism | |
| --- | --- | --- |
|  | Female | Male |
| Amphetamine | 1 | 1 |
| Aripiprazole | 0 | 1 |
| Atomoxetine | 3 | 1 |
| Bupropion | 0 | 1 |
| Buspirone | 1 | 0 |
| Chlorpromazine | 1 | 1 |
| Citalopram | 1 | 0 |
| Clonidine | 3 | 0 |
| Dexmethylphenidate | 0 | 3 |
| Escitalopram | 1 | 1 |
| Fluoxetine | 4 | 2 |
| Fluvoxamine | 0 | 1 |
| Guanfacine | 2 | 1 |
| Lisdexamfetamine | 2 | 0 |
| Methylphenidate | 0 | 5 |
| Oxcarbazepine | 0 | 1 |
| Quetiapine | 2 | 1 |
| Risperidone | 1 | 4 |
| Sertraline | 3 | 4 |
| Topiramate | 0 | 1 |
| Trazodone | 0 | 1 |

A total of 15 autistic female participants and 17 autistic male participants were on one or more psychotropic medications. All TD subjects were confirmed to not be on any psychotropic medications.

Table S2: Mean and Standard Deviation of Phantom Temporal Signal to Noise Ratios

|  | Harvard Trio | Seattle Trio | Seattle Prisma | UCLA Trio | UCLA Prisma | Yale Trio |
| --- | --- | --- | --- | --- | --- | --- |
| Sphere | 122.46 + 5.82 | 155.00 + 8.43 | 178.50^a^ | 154.02 + 7.02 | 178.30^a^ | 182.56 + 4.15 |
| Human 1 | 145.84 + 23.57 | 162.24 + 15.90 | 153.78 + 22.12 | 148.70 + 11.41 | - | 156.23 + 8.44 |
| Human 2 | 167.78 + 11.34 | - | - | - | - | 162.68 + 11.23 |

^a^Only one functional imaging protocol collected, instead of the five collected for the overall multisite project.

Table S3. Peak Coordinates for Altered Whole-Brain Activity to Non-Social Rewards (Male Autism > Male TD)

| Region | L/R | Max *Z* | MNI Peak (mm) | | | Sig # Voxels |
| --- | --- | --- | --- | --- | --- | --- |
|  |  |  | x | y | z |  |
| *Midline Frontal Cluster, d=0.53* |  |  |  |  |  |  |
| Frontal Pole | L | 3.20 | -6 | 60 | 22 | 22 |
| Superior Frontal Gyrus | R | 3.20 | 4 | 48 | 34 | 24 |
| Paracingulate Gyrus | L | 3.49 | -2 | 52 | 18 | 45 |
| Paracingulate Gyrus | R | 3.12 | 2 | 44 | 32 | 15 |
| Anterior Cingulate Gyrus | R | 2.81 | 4 | 38 | 20 | 13 |

Regions were labeled using the Harvard-Oxford atlas at a 50% probabilistic threshold. Left/Right masks excluded the midline, and regions were only listed if they included 10 or more active voxels. TD: typically developing; L: Left; R: Right; MNI: Montreal Neurological Institute.

Table S4. Peak Coordinates for Altered Whole-Brain Activity to Social Rewards (Diagnosis and Sex Main Effects)

| Region | L/R | Max *Z* | MNI Peak (mm) | | | Sig # Voxels |
| --- | --- | --- | --- | --- | --- | --- |
|  |  |  | x | y | z |  |
| Autism > TD |  |  |  |  |  |  |
| *Left Frontal Cluster, d=0.40* |  |  |  |  |  |  |
| Frontal Pole | L | 3.62 | -8 | 68 | 8 | 382 |
| *Left/Midline Frontal Cluster, d=0.63* |  |  |  |  |  |  |
| Frontal Pole | L | 3.05 | -6 | 58 | 16 | 18 |
| Frontal Medial Cortex | L | 3.45 | -6 | 50 | -16 | 52 |
| Anterior Cingulate Gyrus | L | 4.24 | -6 | 40 | 12 | 71 |
| Anterior Cingulate Gyrus | R | 3.49 | 2 | 42 | 0 | 19 |
| Paracingulate Gyrus | L | 3.48 | -2 | 44 | -4 | 166 |
| Paracingulate Gyrus | R | 3.38 | 6 | 38 | 34 | 61 |
| Superior Frontal Gyrus | R | 3.15 | 2 | 46 | 40 | 12 |
| Female > Male |  |  |  |  |  |  |
| *Bilateral Parietal/Occipital Cluster, d=0.40* |  |  |  |  |  |  |
| Precuneus Cortex | L | 2.99 | -6 | -74 | 38 | 54 |
| Precuneus Cortex | R | 3.60 | 12 | -68 | 32 | 104 |
| Lateral Occipital Cortex | R | 2.63 | 20 | -78 | 42 | 11 |

Regions were labeled using the Harvard-Oxford atlas at a 50% probabilistic threshold. Left/Right masks excluded the midline, and regions were only listed if they included 10 or more active voxels. TD: typically developing; L: Left; R: Right; MNI: Montreal Neurological Institute.

Table S5. Peak Coordinates of Activity to Social Rewards.

|  |  | Female Autism | | | | |  | Male Autism | | | | |  | Female TD | | | | |  | Male TD | | | | |
| --- | --- | --- | --- | --- | --- | --- | --- | --- | --- | --- | --- | --- | --- | --- | --- | --- | --- | --- | --- | --- | --- | --- | --- | --- |
| Region | L/R | Max *Z* | MNI Peak (mm) | | | Sig # Voxels |  | Max *Z* | MNI Peak (mm) | | | Sig # Voxels |  | Max *Z* | MNI Peak (mm) | | | Sig # Voxels |  | Max *Z* | MNI Peak (mm) | | | Sig # Voxels |
|  |  |  | x | y | z |  |  |  | x | y | z |  |  |  | x | y | z |  |  |  | x | y | z |  |
| Accumbens | L | 4.89 | -6 | 8 | -6 | 49 |  | - | - | - | - | - |  | 2.85 | -8 | 12 | -6 | 15 |  | 3.04 | -14 | 14 | -8 | 10 |
| Accumbens | R | 3.79 | 12 | 10 | -10 | 28 |  | - | - | - | - | - |  | - | - | - | - | - |  | - | - | - | - | - |
| Amygdala | L | 2.96 | -14 | -4 | -20 | 17 |  | - | - | - | - | - |  | - | - | - | - | - |  | - | - | - | - | - |
| Amygdala | R | 3.24 | 20 | -2 | -12 | 30 |  | - | - | - | - | - |  | - | - | - | - | - |  | - | - | - | - | - |
| Anterior Cingulate Gyrus | L | 3.07 | -2 | 38 | 6 | 54 |  | 4.15 | -6 | 42 | 10 | 94 |  | - | - | - | - | - |  | - | - | - | - | - |
| Anterior Cingulate Gyrus | R | 2.94 | 2 | 42 | 2 | 29 |  | 3.00 | 2 | 38 | -4 | 18 |  | - | - | - | - | - |  | - | - | - | - | - |
| Caudate | L | 3.01 | -8 | 8 | -2 | 10 |  | - | - | - | - | - |  | - | - | - | - | - |  | - | - | - | - | - |
| Central Opercular Cortex | R | 3.38 | 56 | 4 | 2 | 47 |  | - | - | - | - | - |  | - | - | - | - | - |  | - | - | - | - | - |
| Cuneal Cortex | L | 4.27 | -2 | -76 | 28 | 67 |  | - | - | - | - | - |  | - | - | - | - | - |  | - | - | - | - | - |
| Cuneal Cortex | R | 4.25 | 8 | -78 | 28 | 123 |  | - | - | - | - | - |  | - | - | - | - | - |  | - | - | - | - | - |
| Frontal Medial Cortex | L | 3.00 | -2 | 38 | -22 | 31 |  | 3.76 | -6 | 44 | -12 | 58 |  | - | - | - | - | - |  | - | - | - | - | - |
| Frontal Medial Cortex | R | 3.64 | 8 | 46 | -12 | 53 |  | 2.83 | 2 | 44 | -12 | 21 |  | - | - | - | - | - |  | - | - | - | - | - |
| Frontal Pole | L | 3.82 | -46 | 40 | 10 | 417 |  | 2.56 | -2 | 58 | -2 | 10 |  | - | - | - | - | - |  | - | - | - | - | - |
| Frontal Pole | R | 3.13 | 8 | 70 | 10 | 69 |  | - | - | - | - | - |  | - | - | - | - | - |  | - | - | - | - | - |
| Inferior Frontal Gyrus | L | 3.23 | -52 | 32 | -2 | 45 |  | - | - | - | - | - |  | - | - | - | - | - |  | - | - | - | - | - |
| Insula | R | 2.95 | 38 | 2 | -4 | 24 |  | - | - | - | - | - |  | - | - | - | - | - |  | - | - | - | - | - |
| Intracalcarine Cortex | L | 2.99 | -4 | -66 | 10 | 36 |  | - | - | - | - | - |  | - | - | - | - | - |  | - | - | - | - | - |
| Intracalcarine Cortex | R | 4.12 | 12 | -78 | 10 | 97 |  | - | - | - | - | - |  | - | - | - | - | - |  | - | - | - | - | - |
| Lateral Occipital Cortex | L | 3.02 | -26 | -86 | 30 | 23 |  | - | - | - | - | - |  | - | - | - | - | - |  | - | - | - | - | - |
| Lateral Occipital Cortex | R | 3.02 | 24 | -82 | 38 | 63 |  | - | - | - | - | - |  | - | - | - | - | - |  | - | - | - | - | - |
| Lingual Gyrus | R | 4.16 | 10 | -64 | -6 | 232 |  | - | - | - | - | - |  | - | - | - | - | - |  | - | - | - | - | - |
| Orbital Frontal Cortex | L | 3.83 | -22 | 8 | -18 | 30 |  | - | - | - | - | - |  | - | - | - | - | - |  | - | - | - | - | - |
| Paracingulate Gyrus | L | 3.40 | -4 | 52 | 6 | 90 |  | 3.91 | -2 | 52 | -4 | 84 |  | - | - | - | - | - |  | - | - | - | - | - |
| Paracingulate Gyrus | R | 3.04 | 4 | 50 | -2 | 56 |  | 2.91 | 2 | 48 | -6 | 17 |  | - | - | - | - | - |  | - | - | - | - | - |
| Parietal Operculum Cortex | R | 2.83 | 48 | -30 | 20 | 23 |  | - | - | - | - | - |  | - | - | - | - | - |  | - | - | - | - | - |
| Planum Polare | R | 3.78 | 58 | 2 | 0 | 13 |  | - | - | - | - | - |  | - | - | - | - | - |  | - | - | - | - | - |
| Precentral Gyrus | R | 2.78 | 58 | 8 | 8 | 13 |  | - | - | - | - | - |  | - | - | - | - | - |  | - | - | - | - | - |
| Precuneus Cortex | L | 3.30 | -6 | -74 | 38 | 115 |  | - | - | - | - | - |  | - | - | - | - | - |  | - | - | - | - | - |
| Precuneus Cortex | R | 3.51 | 2 | -70 | 28 | 103 |  | - | - | - | - | - |  | - | - | - | - | - |  | - | - | - | - | - |
| Putamen | L | 3.78 | -14 | 6 | -8 | 34 |  | - | - | - | - | - |  | - | - | - | - | - |  | - | - | - | - | - |
| Putamen | R | 3.77 | 16 | 6 | -10 | 67 |  | - | - | - | - | - |  | - | - | - | - | - |  | - | - | - | - | - |
| Subcallosal Cortex | R | 2.63 | 2 | 12 | -10 | 19 |  | - | - | - | - | - |  | - | - | - | - | - |  | - | - | - | - | - |
| Superior Temporal Gyrus | R | 3.80 | 62 | 0 | -4 | 73 |  | - | - | - | - | - |  | - | - | - | - | - |  | - | - | - | - | - |
| Temporal Pole | R | 2.90 | 52 | 10 | -8 | 13 |  | - | - | - | - | - |  | - | - | - | - | - |  | - | - | - | - | - |

Regions were labeled using the Harvard-Oxford atlas at a 50% probabilistic threshold. Left/Right masks excluded the midline, and regions were only listed if they included 10 or more active voxels. TD: typically developing; L: Left; R: Right; MNI: Montreal Neurological Institute.

*Supplementary Figures*

Figure S1: Activity within each group to social rewards. TD: typically developing; L, left.
